# Bioprinted human skin equivalent for evaluating non-invasive monopolar radiofrequency treatment: a novel approach

**DOI:** 10.1007/s10103-026-04908-2

**Published:** 2026-06-10

**Authors:** Hye Guk Ryu, Chaeeun Kim, Nark-kyoung Rho, Jumi Hong, Jinyoung Park, Wanil Kim, Jiyeon Na, Sangjune Kim, Soo Il Chun, Hwa-Rim Lee, Sungjune Jung, Sung Bin Cho

**Affiliations:** 1 Clinical Development Team, Cynosure Lutronic Corporation, Goyang, Korea, Republic of; 2 Department of Materials Science & Engineering, Pohang University of Science and Technology (POSTECH), Pohang, Korea, Republic of; 3 Leaders Aesthetic Laser & Cosmetic Surgery Center, Seoul, Korea, Republic of; 4 Department of Biochemistry and Institute of Medical Science, School of Medicine, Gyeongsang National University, Jinju, Korea, Republic of; 5 Department of Biological Sciences and Biotechnology, Chungbuk National University, Cheongju, Korea, Republic of; 6 Chunsooil Skin Clinic, Seoul, Korea, Republic of; 7 Department of Pharmacology, School of Medicine, Kyungpook National University, Daegu, Korea, Republic of; 8 Yonsei Seran Dermatology and Laser Clinic, Seoul, Korea, Republic of

**Keywords:** 3D dermis model, Bioprinting, Non-invasive monopolar radiofrequency, Collagen, Dermal fibroblast, Wound healing, Anti-melanogenesis, Cell viability

## Abstract

To establish a standardized 3D human dermis model for evaluating the biological and mechanical effects of non-invasive monopolar radiofrequency (NMRF) treatment. Collagen-based 3D dermal constructs with human dermal fibroblasts were fabricated via bioprinting and treated with NMRF. Histology, immunofluorescence, SEM, and compression tests were conducted to assess tissue remodeling. Conditioned media from treated constructs were applied to fibroblasts, melanocytes, and keratinocytes to evaluate paracrine effects on migration, pigmentation, and oxidative stress. NMRF treatment led to increased fibroblast activity and cellular enlargement, along with the formation of a denser and more organized collagen network, as demonstrated by histological and SEM analyses. Immunofluorescence staining revealed a marked increase in type I collagen deposition surrounding activated fibroblasts. Biomechanical testing showed a significant rise in compressive modulus and reduction in tissue dimensions, indicating enhanced dermal stiffness and matrix remodeling. Furthermore, conditioned media derived from NMRF-treated constructs significantly promoted fibroblast migration in wound healing assays, suppressed the expression of melanogenesis-related genes (MITF, tyrosinase, Trp-1, and Trp-2) in α-MSH-stimulated B16F10 melanoma cells, and improved the survival of keratinocytes exposed to oxidative stress (H₂O₂). This 3D dermis model enables reproducible assessment of NMRF-induced dermal remodeling. NMRF improves dermal structure and modulates paracrine signals, suggesting therapeutic potential in skin tightening, pigmentation control, and tissue regeneration.

**Supplementary Information:**

The online version contains supplementary material available at 10.1007/s10103-026-04908-2.

## Introduction

The skin serves as the primary barrier against environmental insults and plays crucial roles in protection, thermoregulation, immune defense, and homeostasis. The dermis, which constitutes the structural foundation of the skin, is primarily composed of fibroblasts embedded within a collagen-rich extracellular matrix (ECM) [1]. Intrinsic aging and extrinsic stressors contribute to fibroblast dysfunction, leading to decreased collagen production, dermal atrophy, and increased skin laxity [2]. These age-related changes have driven the development of therapeutic interventions aimed at restoring collagen homeostasis and improving skin biomechanical properties.

Non-invasive monopolar radiofrequency (NMRF) has emerged as a promising modality for dermal remodeling and skin tightening [3–5]. By delivering controlled electromagnetic energy, NMRF induces volumetric heating of the dermis, which leads to fibroblast activation and neocollagenesis. Clinical studies have demonstrated improvements in skin elasticity and wrinkle reduction following NMRF treatment [6, 7]. However, the precise mechanisms underlying these effects remain unclear. A critical challenge in evaluating NMRF efficacy is the lack of standardized ex vivo or in vitro models that accurately recapitulate the structural and physiological properties of human skin. Current assessment methodologies primarily rely on in vivo studies, which, while informative, are limited by inter-individual variability, ethical considerations, and technical constraints in monitoring real-time tissue responses.

To address these limitations, we introduced a three-dimensional (3D) bioengineered dermal tissue model as a reproducible platform for investigating the biological and mechanical effects of NMRF. We fabricated collagen-based 3D structures using standardized tissue culture inserts and bioprinting technology. This approach reduced sample variation—a common drawback in 3D model-based testing—and ensured reliability across repeated testing [8, 9]. Use of this model enabled the controlled evaluation of fibroblast responses, collagen remodeling, and ECM structural changes post-treatment. We characterized the impact of NMRF on dermal architecture and mechanical properties using a combination of histological, ultrastructural, and biomechanical analyses.

This study aimed to establish and validate a 3D in vitro dermis model as an advanced tool for assessing the efficacy of NMRF in inducing collagen remodeling and improving dermal biomechanics. These findings provide mechanistic insights into NMRF-mediated skin rejuvenation and offer a standardized model for future investigations into radiofrequency-based dermatological interventions.

## Materials and methods

### Preparation of the 3D dermis model

To develop a biomimetic human dermal equivalent, a microextrusion-based fabrication process was employed with modifications to optimize dermal matrix composition. The medical grade type I collagen (Dalim Tissen, Republic of Korea) was dissolved in 0.1% acetic acid to obtain a 0.75% (w/v) solution. To facilitate fibroblast proliferation within the matrix, 10× DMEM/F-12 medium (Gibco, Grand Island, NY, USA), penicillin (100 U/mL), and streptomycin (100 µg/mL) (HyClone, Logan, UT, USA) were incorporated into the solution. The collagen matrix was neutralized using a reconstitution buffer containing NaOH, NaHCO₃, and HEPES, adjusting the final collagen concentration to 0.6% (w/v). Neonatal human dermal fibroblasts (NHDF, Gibco) were enzymatically detached using trypsin-EDTA (Gibco) and resuspended in the bioink at a density of 2.5 × 10⁵ viable cells/mL before bioprinting.

### Bioprinting and tissue culture

The bioink, composed of 0.6% collagen and NHDF cells (2.5 × 10⁵ cells/mL, passage 6–10), was dispensed directly into customized tissue culture inserts with 3.0 μm pore polycarbonate membranes (Corning Inc., Corning, NY, USA) using a pneumatic extrusion bioprinter (350PC, Musashi Engineering, Japan) fitted with a 24-gauge needle at a controlled pressure of 10 kPa. The printed structures were initially maintained at 4 °C before undergoing crosslinking at room temperature, followed by incubation at 37 °C in a 5% CO₂ humidified environment. The constructs were then submerged in a fibroblast culture medium (fibroblast expansion basal medium supplemented with LSGS and antibiotics), which was refreshed every 2 to 3 days [9].

### Application of non-invasive monopolar radiofrequency (NMRF) devices and surface temperature measurement

An NMRF device (XERF, Cynosure Lutronic Inc., Goyang, Korea) was utilized to deliver controlled monopolar RF energy to the 3D dermis model. The 3D dermis constructs, which had been maintained in culture for 8 days after printing, were placed on a return pad before undergoing treatment with the Deep mode at energy level 5. RF energy was delivered as a single shot, with the treatment tip placed in direct contact with the surface of the 3D dermis constructs. The exposure duration for each construct was less than 2 s. During the procedure, an infrared thermal imaging camera (FLIR A325, FLIR System Inc., Wilsonville, OR, USA) was used to monitor real-time temperature changes across the surface of the constructs, assessing the efficiency of RF energy delivery. Following the treatment, the constructs were returned to culture and incubated in a fibroblast culture medium, with the medium being refreshed every 2 to 3 days.

### Morphological observations

To investigate the ultrastructural changes induced by NMRF treatment, scanning electron microscopy (SEM) analysis was performed. For sample fixation, the 3D dermis constructs were initially immersed in 2.5% glutaraldehyde (Sigma-Aldrich, St. Louis, MO, USA) in 0.1 M phosphate-buffered saline (PBS) at 4 °C for 24 h. The samples were then rinsed three times with PBS and post-fixed in 1% osmium tetroxide (OsO₄) for 1 h at room temperature. Following fixation, the constructs were dehydrated through a graded ethanol series (30%, 50%, 70%, 90%, and 100%) and subjected to critical point drying (LEICA EM CPD300; Leica, Wien, Austria). Dried specimens were mounted on aluminum stubs and coated with a thin layer of platinum (5 nm) using a sputter coater (Leica EM ACE600, Leica Microsystems, Wetzlar, Germany) to enhance conductivity. SEM imaging was conducted using a Sigma 360 scanning electron microscope (Carl Zeiss, Oberkochen, Germany) under an accelerating voltage of 2 kV. High-resolution images were acquired to assess ECM organization, collagen fiber morphology, and cellular interactions within the 3D dermis model.

### Histological and immunohistochemical analyses

For histological evaluation, 3D dermis constructs were collected 2 weeks after RF treatment and were subjected to cryopreservation-based processing. Following completion of the culture period, constructs were equilibrated in 30% sucrose-PBS solution, fixed in 4% paraformaldehyde, and embedded in an optimal cutting temperature (OCT) compound (CellPath Ltd., Newtown, UK). Cryosections (15 μm) were prepared using a cryotome (CM1860, Leica Biosystems, Nussloch, Germany) and subsequently stained with hematoxylin and eosin (Dako, Carpinteria, CA, USA) to assess tissue architecture.

Immunohistochemistry was performed on cryosectioned tissues using the following primary antibodies: anti-collagen type I (Abcam, Cambridge, UK). Alexa Fluor^®^ 488-conjugated goat anti-rabbit IgG (Invitrogen, Camarillo, CA, USA) was used as the secondary antibody, and nuclei were counterstained with Hoechst 33,342 (Invitrogen). Images were acquired to assess newly synthesized collagen using a fluorescence microscope (Ti2-E; Nikon Corp., Tokyo, Japan).

### Mechanical characterization of the 3D dermis model

To evaluate the biomechanical properties of the engineered dermal constructs, axial compression testing was performed 2 weeks after RF treatment using a rheometer (Discovery HR 20, TA Instruments, New Castle, DE, USA). The dermal matrices, shaped into uniform discs (12 mm in diameter), were positioned between the lower platen (25 °C) and a parallel spindle (20 mm diameter). A controlled compression rate of 20.0 μm/s was applied for 500 s, and the compressive modulus was determined by analyzing the linear region of the stress-strain curve.

### Cell culture and treatments

Human dermal fibroblasts (Hs68), B16F10, and HaCaT cells were kindly provided by Dr. K.T. Kim and Dr. J. Lee of POSTECH. Hs68, HaCaT, and B16F10 cells were maintained in DMEM supplemented with GlutaMAX™ (Cat. no. 10566024, Gibco), 10% Fetal Bovine Serum (Premium Imported Origin from Canada, Cat. no. 12483020, Gibco), and Penicillin–Streptomycin (10,000 U/mL, Cat. no. 15140122, Thermo Fisher Scientific, Waltham, MA, USA) [10]. All experiments were conducted using cells that had not exceeded passage 10 after being received.

### Scratch wound assay

To investigate the paracrine effect of the 3D dermis model, a wound healing assay was performed. When Hs68 cells reached 100% confluency, a scratch was introduced into each culture using an SPLScar™ Scratcher (SPL Life Sciences, Republic of Korea), followed by three washes with PBS to remove detached cells. The cultures were then treated with one of the following conditions: untreated cells that were not exposed to α-MSH or H₂O₂ stimulation (Untreated), conditioned medium from untreated 3D dermis models (Vehicle), or conditioned medium from NMRF-treated 3D dermis models (NMRF). Conditioned medium (CM) was collected, filtered and centrifuged at 300 x g for 5 min to remove cell debris. The wound area was captured using an inverted microscope immediately after CM application and again after 48 h to assess the healing response.

### RNA isolation, cDNA synthesis, and quantitative RT-PCR

Total RNA was extracted using the RNeasy Plus Mini Kit (Qiagen, Valencia, CA, USA) following the manufacturer’s protocol. cDNA was synthesized from 1 µg of total RNA using PrimeScript™ RT Master Mix (Takara, Otsu, Japan) under standard reaction conditions. Quantitative real-time PCR (qRT-PCR) was performed using SYBR™ Green PCR Master Mix (Applied Biosystems, Foster City, CA, USA) with gene-specific primers. The thermal cycling conditions included an initial denaturation at 95°C, followed by 40 cycles of 95°C for 15 s and 60°C for 1 min, with a final melt curve analysis to confirm specificity [11]. Relative gene expression was calculated using the 2^−ΔΔCt method, normalized to housekeeping genes. Primer sequences used are listed below: Mitf: Forward 5’-AGC GTG TAT TTT CCC CAC AG-3’ and Reverse 5’-TAG CTC CTT AAT GCG GTC GT-3’, Trp-1: Forward 5’-ACT TCA CTC AAG CCA ACT GC-3’ and Reverse 5’-AGC TTC CCA TCA GAT GTC GT-3’, Trp-2: Forward 5’-GCT CCA AGT GGC TGT AGA CC-3’ and Reverse 5’-AAT GCA GTG GCT TGA AAA TC-3’, Tyrosinase: Forward 5’-GAC GGT CAC TGC ACA CTT TG-3’ and Reverse 5’-GCC ATG ACC AGG ATG AC-3’, and Gapdh: Forward 5’-ACC ACA GTC CAT GCC ATC AC-3’ and Reverse 5’-TCC ACC ACC CTG TTG CTG TA-3’.

### Cell viability assay

Cell viability was assessed using the AlamarBlue™ assay (Thermo Fisher Scientific). Cells were seeded in 96-well plates at a density of 5,000 cells per well and allowed to adhere overnight. The next day, the culture medium was supplemented with treatment conditions, and cells were incubated for 24 h. Following treatment, AlamarBlue™ reagent (1:10 dilution in culture medium) was added to each well and incubated at 37 °C for 2 h. Absorbance was measured at 570 nm and 600 nm using a microplate reader (Infinite^®^ 200 PRO, TECAN, Männedorf, Switzerland), and cell viability was determined based on the relative absorbance values [12]. In parallel, B16F10 cells were also treated with the corresponding CM for 2 days to evaluate the paracrine effects of the 3D dermis model on melanocytic cells.

### Statistical analysis

Statistical analyses were conducted using GraphPad Prism 9 software (GraphPad Software, San Diego, CA, USA). Data are presented as mean ± standard error of the mean (SEM), and statistical significance was determined using an unpaired two-tailed Student’s t-test or one-/two-way ANOVA followed by Bonferroni post-hoc multiple comparison tests. A *p*-value of < 0.05 was considered statistically significant.

## Results

### Development and validation of a 3D dermis model for NMRF evaluation

**Fig. 1:**
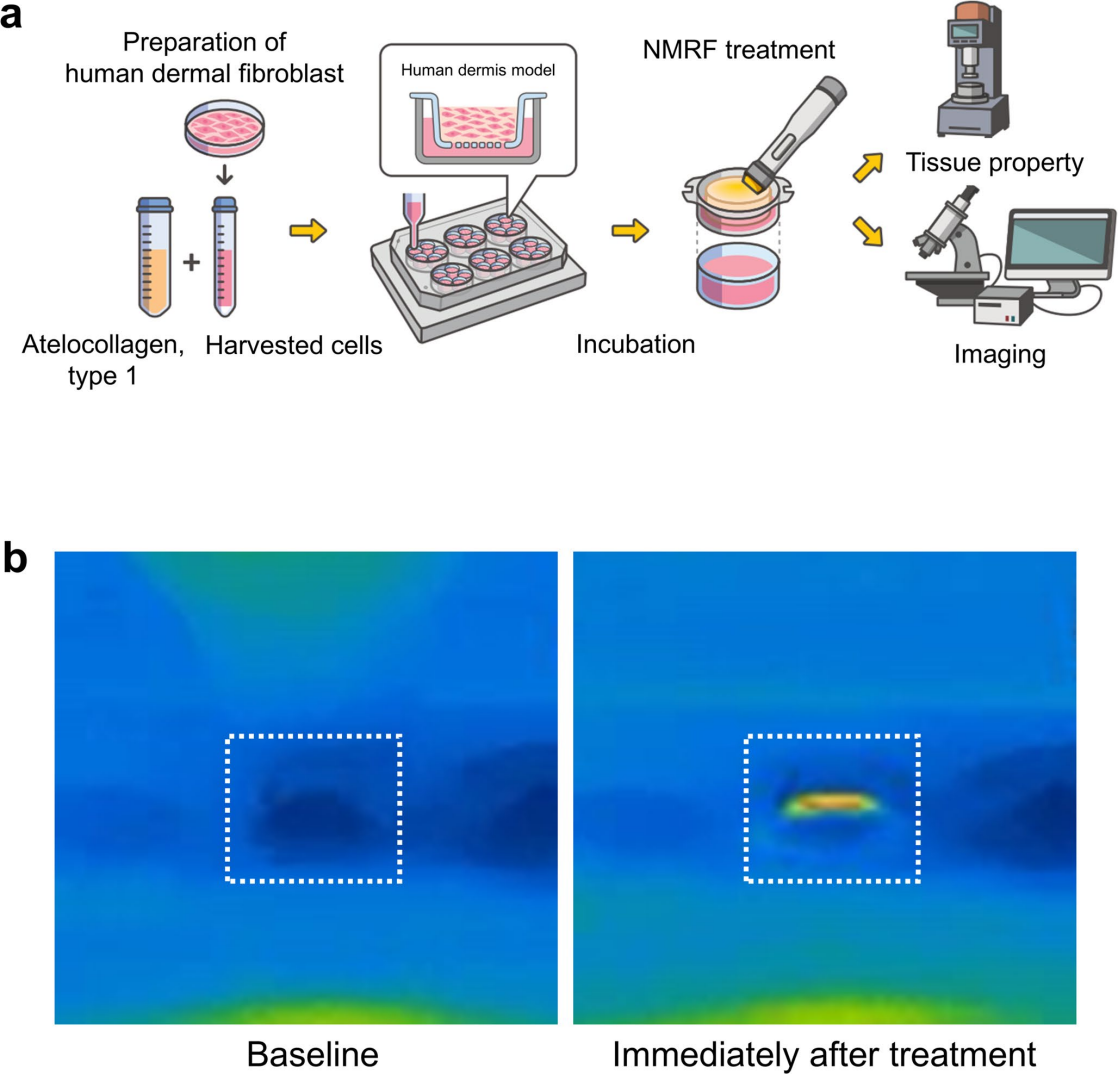
Schematic representation of 3D dermis model fabrication and experimental setup. (**a**) Illustration of the biofabrication process, depicting human dermal equivalent construction, culture, and subsequent NMRF application. (**b**) Infrared thermographic imaging of the dermis model before and after NMRF application, demonstrating effective heat transmission to the tissue. Dotted lines indicate the 3D dermis model placed on the return pad

To assess the biological effects of NMRF treatment, we developed a three-dimensional (3D) bioengineered dermis model that recapitulated the structural and cellular properties of the human dermis (Fig. 1a). The constructs were fabricated using collagen-based bioink containing NHDF and maintained under optimized culture conditions [9]. Upon NMRF application, infrared thermographic imaging demonstrated efficient heat delivery with a controlled temperature increase at the treatment site, suggesting effective energy transmission to the dermis construct (Fig. 1b).

### Histological and ultrastructural analyses of the NMRF-treated dermis model

**Fig. 2:**
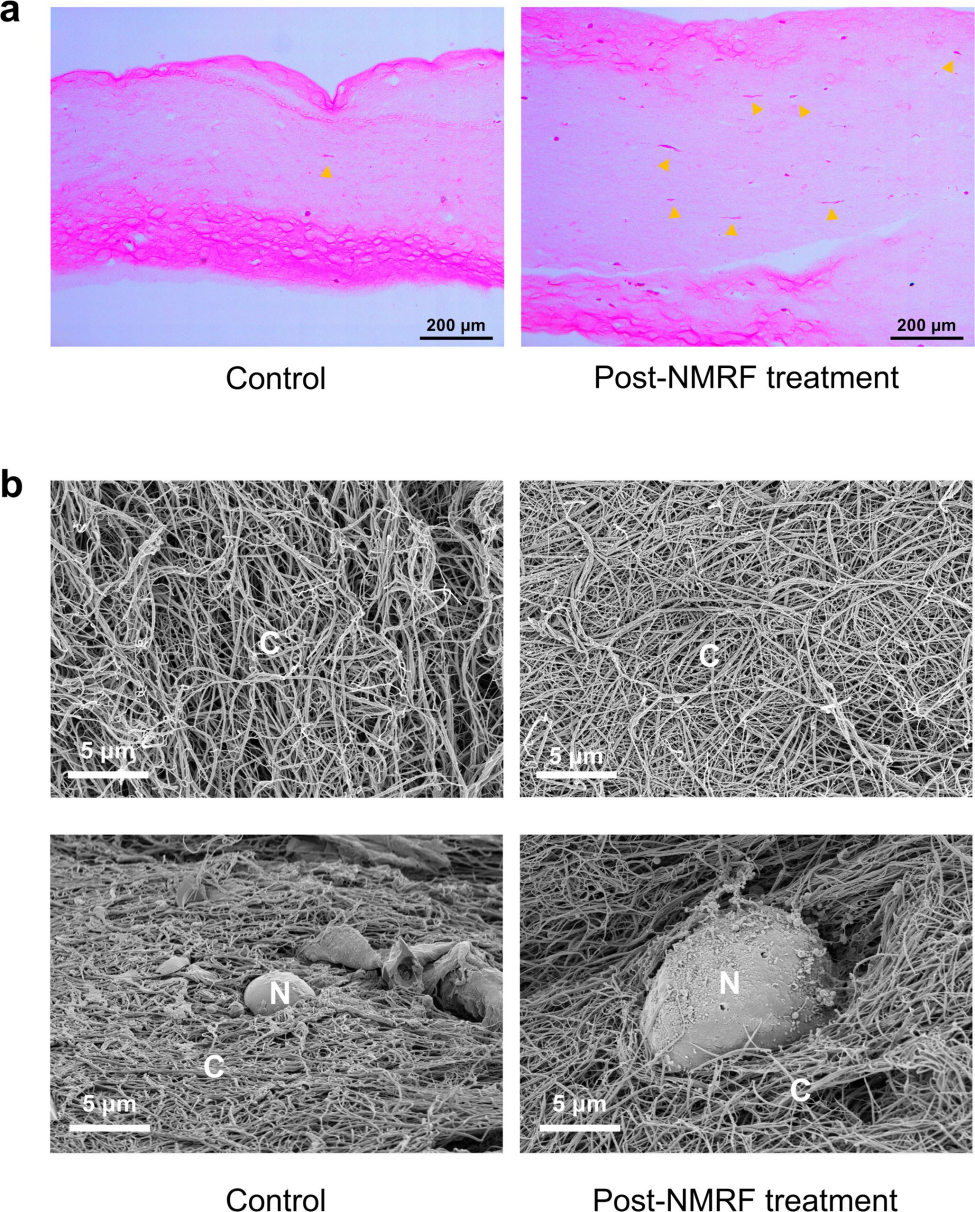
Histological and ultrastructural analyses of NMRF-treated 3D dermis constructs. (**a**) H&E staining of untreated and NMRF-treated constructs, showing increased fibroblast density post-treatment. Enlarged fibroblasts are indicated by arrowheads. (**b**) SEM images demonstrating enhanced collagen network formation and fibroblast enlargement following NMRF application. C, collagen; N, nucleus

**Fig. 3:**
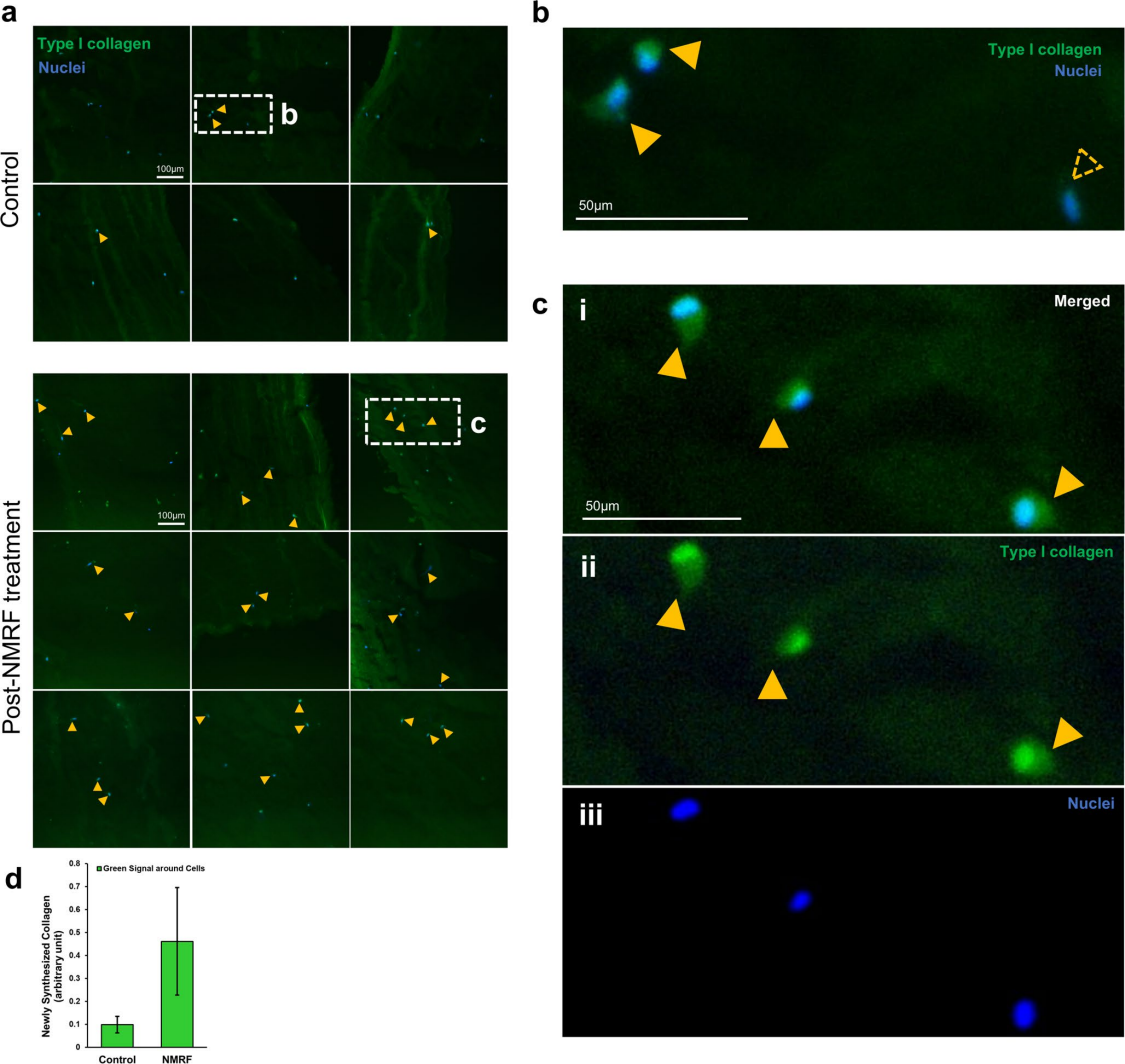
Immunofluorescent analysis of newly synthesized collagen in NMRF-treated 3D dermis constructs. (**a**) Immunofluorescent staining using a type I collagen (COL1) antibody and Hoechst 33342 nuclear staining reagent. All images in panel (a) were acquired at the same magnification (scale bar: 100 μm). Arrowheads indicate newly synthesized collagen surrounding fibroblasts in NMRF-treated constructs. (**b**) Magnified image of the distribution of fibroblasts and surrounding newly synthesized collagen (arrowheads) and non-synthesized cells (arrowheads, dashed) in the control group. (**c**) Magnified image of the distribution of fibroblasts and surrounding newly synthesized collagen (arrowheads) in NMRF-treated constructs. (**d**) Quantification of newly synthesized collagen patterns around fibroblasts. Quantification was performed by comparing the number of cells with collagen distribution (green) to the total number of cells (blue). *n* = 15. Mann–Whitney U test: U = 211.5, *p* = 3.33 × 10^− 5^ (*p* < 0.0001)

Hematoxylin and eosin staining revealed a well-organized fibroblast-populated ECM, with an increased cellular density following NMRF treatment (Fig. 2a). Scanning electron microscopy (SEM) analysis demonstrated enhanced collagen deposition and increased fibroblast size in NMRF-treated constructs, indicating increased metabolic activity and ECM remodeling (Fig. 2b). Based on the presence of collagen as shown in the structural analyses, we evaluated collagen production by fibroblasts, which primarily promotes ECM secretion and remodeling. Immunohistochemical staining for type I collagen (COL1) revealed a significant increase in the number of cells containing newly generated type I collagen around dermal fibroblasts following NMRF exposure, supporting the hypothesis that NMRF promotes collagen biosynthesis and dermal remodeling (Fig. 3a-d).

### Biomechanical assessment of the NMRF-treated 3D dermis models

**Fig. 4:**
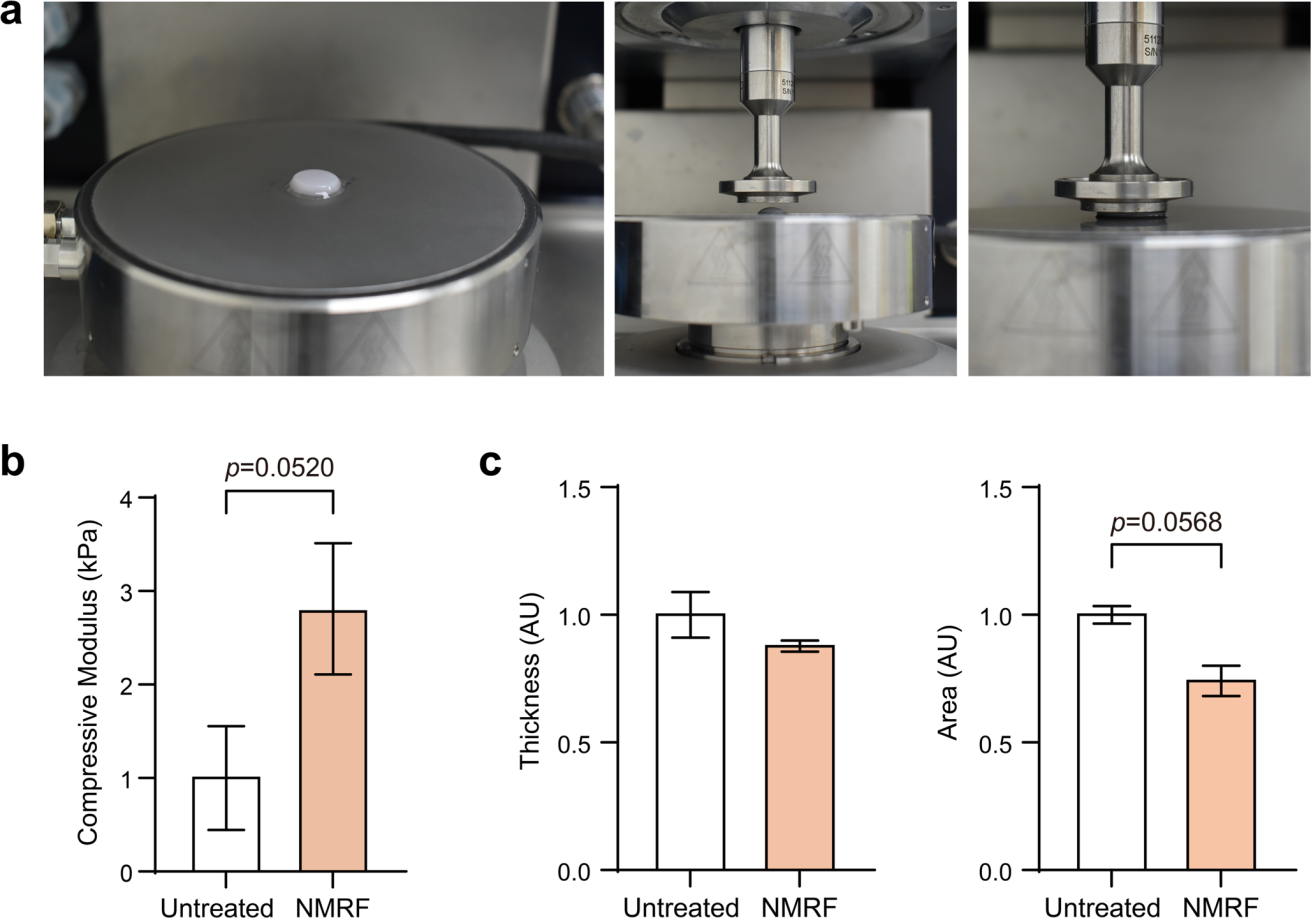
Biomechanical properties of NMRF-treated dermis constructs. (**a**, **b**) Compression test using rotational rheometer demonstrating a significant increase in the compressive modulus of NMRF-treated constructs, indicative of enhanced tissue stiffness. (**c**) Analysis of 3D dermis construct thickness and area reduction after treatment

To evaluate the changes in the mechanical properties of the dermis model after treatment, we conducted a compression test (Fig. 4a, b). The compressive moduli of the NMRF-treated constructs were higher than those of the untreated controls, reflecting enhanced tissue stiffness and structural integrity. These findings indicate that NMRF application improved mechanical properties, likely owing to increased collagen deposition and matrix remodeling. Additionally, analysis of the tissue area and thickness revealed a reduction in both parameters following NMRF treatment, indicating that tissue contraction occurred owing to fibroblast activation during the tissue repair process (Fig. 4c).

### Paracrine effects of NMRF-treated dermal constructs on skin cells

**Fig. 5:**
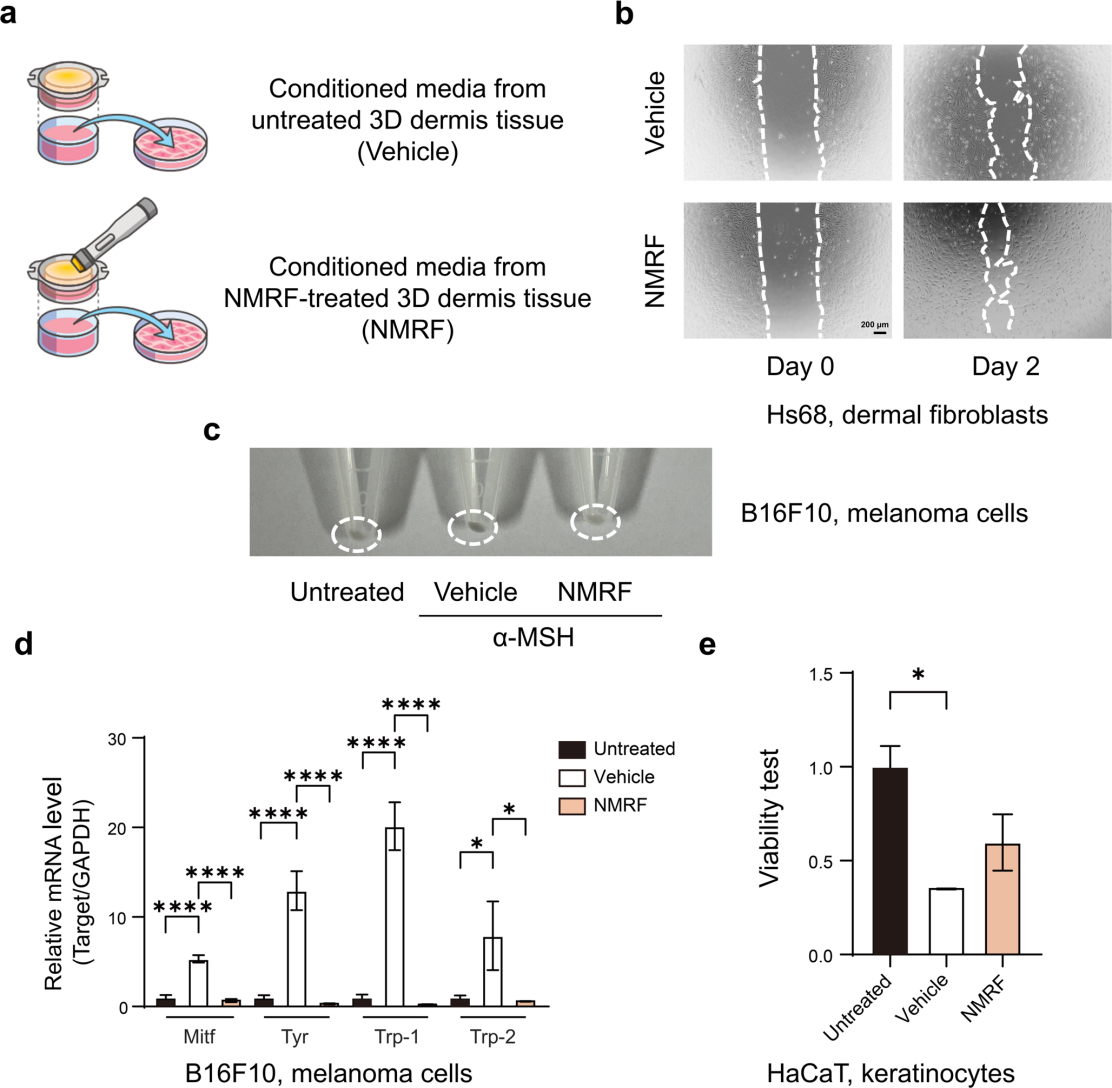
Paracrine effects of NMRF-treated dermis constructs on fibroblast migration, melanogenesis and oxidative stress response. (**a**) Schematic representation of the paracrine effect experiment, illustrating the workflow using conditioned media from untreated or NMRF-treated dermis constructs applied to fibroblasts, melanocytes, or keratinocytes. (**b**) Migration assay of Hs68 fibroblasts showing enhanced wound closure upon treatment with NMRF-conditioned media. (**c**, **d**) B16F10 melanoma cells stimulated with α-MSH and treated with NMRF-conditioned media exhibited visible pellet whitening, along with downregulation of melanogenesis-associated markers (MITF, tyrosinase, Trp-1, Trp-2). Cell pellets (white dotted circles) showing color variations under different treatment conditions. (**e**) Keratinocytes subjected to oxidative stress (H₂O₂ exposure) demonstrated increased survival rates when treated with NMRF-conditioned media, suggesting potential protective effects against ROS-induced damage

To explore the broader biological effect of NMRF treatment beyond direct fibroblast stimulation, we investigated whether NMRF-modulated dermis constructs exerted paracrine effects on various skin cell types [13–15]. Given that native skin comprises a diverse population of cells, including fibroblasts, melanocytes, and keratinocytes, we aimed to assess how factors secreted from treated dermis constructs influenced cellular responses. To investigate whether NMRF-modulated dermis constructs exerted paracrine effects on the surrounding skin cells, conditioned media from NMRF-treated constructs were collected and applied to various cell types (Fig. 5a).

Migration assays using HDFs (Hs68) demonstrated enhanced wound closure upon treatment with NMRF-conditioned media, suggesting the presence of secretory factors that promoted fibroblast motility and tissue repair (Fig. 5b). Furthermore, when murine melanoma cells (B16F10) were stimulated with α-melanocyte-stimulating hormone (α-MSH) and subsequently treated with NMRF-conditioned media, pellet whitening was observed, accompanied by a significant reduction in the expression of melanogenic markers MITF, tyrosinase, Trp-1, and Trp-2 (Fig. 5c and d). This suggests that the factors secreted by NMRF-treated fibroblasts reduced melanogenesis.

Lastly, oxidative stress-induced keratinocytes (H_2_O_2_-treated) exhibited increased cell survival following exposure to NMRF-conditioned media, indicating potential cytoprotective effects against reactive oxygen species (ROS)-induced damage (Fig. 5e). Collectively, these findings suggest that NMRF-mediated dermal remodeling may have broad implications in skin regeneration, anti-pigmentation effects, and oxidative stress resilience.

## Discussion

This study presents a novel in vitro 3D dermis model specifically developed to evaluate the biological effects of NMRF. Traditional in vivo models of RF-induced skin remodeling have inherent limitations, including inter-individual variability, ethical concerns, and technical constraints. When skin cells are mixed with collagen and fabricated using manual methods such as pipetting, it is difficult to ensure consistent injection volumes between samples due to hydrogel viscosity and handling difficulties. Furthermore, the asymmetric meniscuses that inevitably occur during manual fabrication lead to sample asymmetry after gelation. This causes significant experimental errors not only in sample-sample variation but also in the mechanical property measurement process demonstrated in this study. On the other hand, bioprinting technology inherently guarantees reproducibility and precise control, thereby reducing sample variation and experimental errors and resolving the aforementioned problems. Through the use of the bioprinted human dermis model, we established a controlled and reproducible platform that enabled precise histological, ultrastructural, and biomechanical analyses after NMRF treatment.

This model provides critical insights into RF-mediated dermal remodeling and serves as a valuable foundation for expanding the applications of NMRF in dermatology.

A major finding of this study was that NMRF significantly enhanced dermal remodeling by stimulating fibroblast activity and increasing collagen density. Histological and ultrastructural assessments demonstrated a more compact and organized collagen network, coupled with an increase in fibroblast size, indicating heightened cellular activity and ECM production. These changes were further supported by the evaluation of the mechanical properties of the skin tissues, which revealed a significant increase in the compressive modulus and a reduction in the tissue area and thickness after treatment. These data suggest that NMRF contributes to dermal tightening and mechanical reinforcement by promoting collagen compaction and matrix reorganization. Of note, although the collagen network appeared denser following NMRF treatment, the thickness of individual collagen fibers measured on SEM images was not significantly altered. This is likely because lateral fibril fusion into thicker bundles requires regulatory matrix components such as small leucine-rich proteoglycans and minor collagens, which are not fully reconstituted in this simplified collagen–fibroblast model, and because the two-week observation period may be insufficient for such fibril maturation to occur. Thus, our findings indicate that NMRF primarily promotes collagen deposition and network densification rather than fiber thickening per se in this model.

In addition to its direct impact on the dermal structure, NMRF treatment also influenced the paracrine signaling environment, affecting multiple skin cell types. Conditioned media from NMRF-treated constructs significantly altered fibroblast migration, melanogenesis, and the oxidative stress response in keratinocytes. These findings indicate that NMRF modulated the dermal secretome, leading to secondary effects that extended beyond direct fibroblast stimulation.

Notably, NMRF-conditioned media suppressed melanin synthesis in α-MSH-stimulated B16F10 melanoma cells, evidenced by reduced expression of MITF, tyrosinase, Trp-1, and Trp-2. This suggests that NMRF may exert a depigmenting effect, which may have therapeutic implications for hyperpigmentation disorders. Although previous studies have primarily focused on RF-induced neocollagenesis [16, 17], our findings highlight that NMRF-mediated dermal remodeling may also influence epidermal pigmentation pathways via fibroblast-derived signaling molecules.

Additionally, NMRF-conditioned media enhanced fibroblast migration, suggesting a potential role in wound healing and tissue regeneration. Given that fibroblast motility is essential for dermal repair and ECM remodeling [18, 19], these results support the hypothesis that NMRF has therapeutic applications beyond aesthetic skin tightening, particularly in regenerative dermatology. Furthermore, the cytoprotective effects observed in keratinocytes following exposure to NMRF-conditioned media indicate that RF-induced secretome alterations may confer resilience against oxidative stress, which is a key factor in photoaging and inflammatory skin conditions. An important consideration in this study is the introduction of a dermis model that excluded the influence of the epidermal layer. We opted for a dermis-only model for two reasons. First, the cooling mechanism inherent in NMRF devices minimizes epidermal thermal effects [20, 21], allowing us to focus specifically on the direct impact of RF energy on the dermis. Second, the inclusion of an epidermal layer introduces significant variability between constructs, as epidermal stratification and differentiation are highly sensitive to culture conditions. Such heterogeneity can compromise experimental reproducibility, making it challenging to achieve standardized assessments of NMRF efficacy. Given these factors, a dermis-only model ensures a controlled and reproducible system for evaluating RF-induced dermal remodeling, while minimizing inter-model variation.

However, a key limitation of current bioprinted skin models, including those used in this study, is the difficulty in achieving a fully developed and reproducible vascular network. Despite the progress in skin tissue engineering, the bioprinting of functional blood and lymphatic vessels remains technically difficult [22]. Because the vascular system plays a critical role in heat dissipation and metabolic responses, its absence may result in inaccurate simulations of energy-based treatment outcomes. This is particularly relevant for therapies such as NMRF or lasers, where thermal dynamics and tissue perfusion significantly affect the efficacy and safety. Although dermis-only bioprinted models offer clear advantages in terms of standardization and reproducibility, these vascular limitations should be considered when interpreting treatment responses or extrapolating preclinical results to clinical scenarios.

Our findings suggest that NMRF treatment has potential benefits beyond skin tightening such as wound healing, pigmentation modulation, and oxidative stress resistance. Future studies should incorporate vascularized coculture systems with keratinocytes and melanocytes to better mimic the native skin microenvironment and enhance the predictive power of preclinical testing platforms for dermatological research and regenerative medicine.

## Supplementary Information

Below is the link to the electronic supplementary material.

Supplementary Material 1

## Data Availability

The datasets generated and/or analyzed during the current study are available from the corresponding author upon reasonable request.
