# Supplementary figures and images for: Bioprinted human skin equivalent for evaluating non-invasive monopolar radiofrequency treatment: a novel approach
Source: Lasers Med Sci. 2026 Jun 10;41(1):111. doi: 10.1007/s10103-026-04908-2 (PMC13253598; doi:10.1007/s10103-026-04908-2)

a

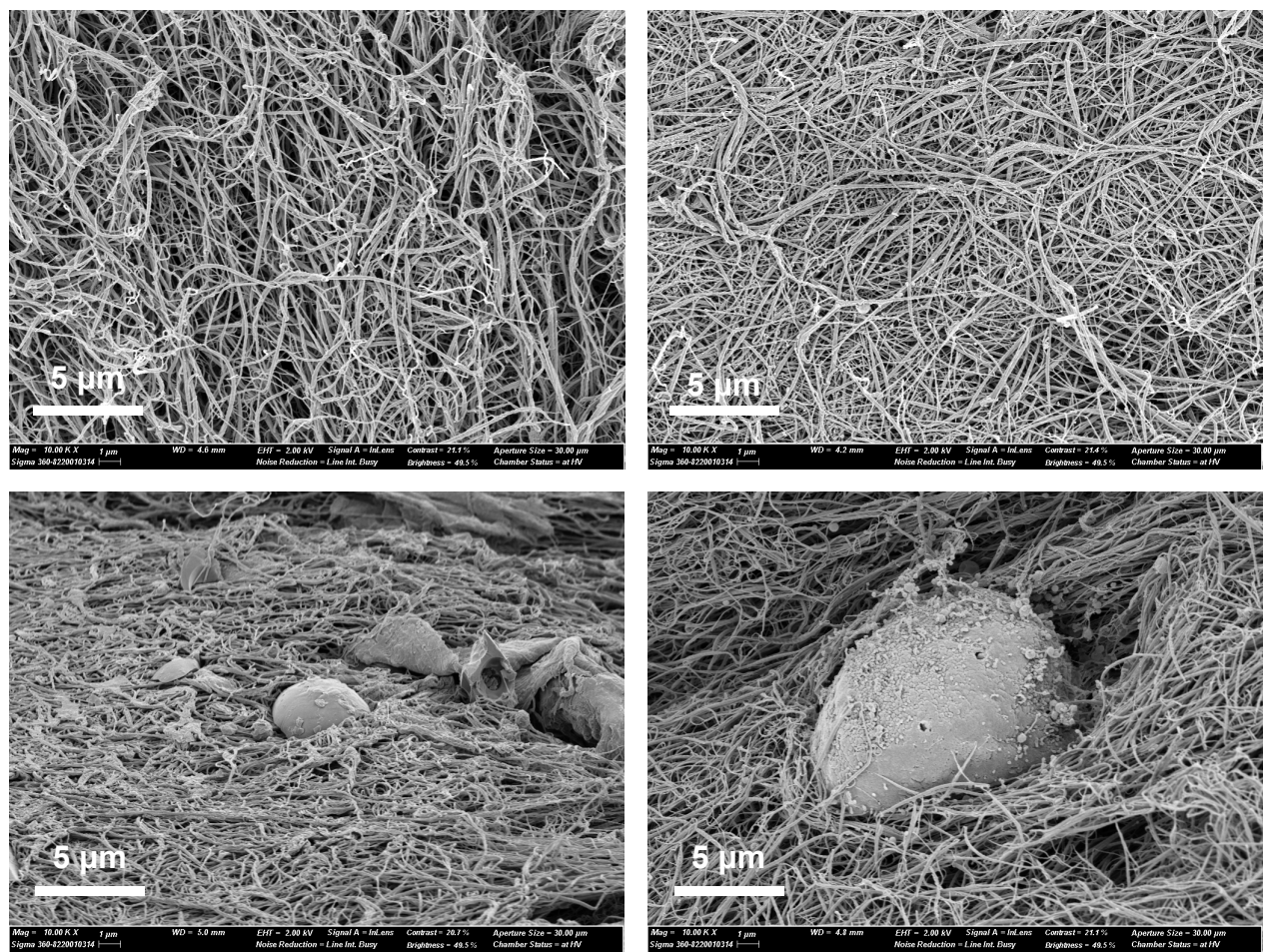

b

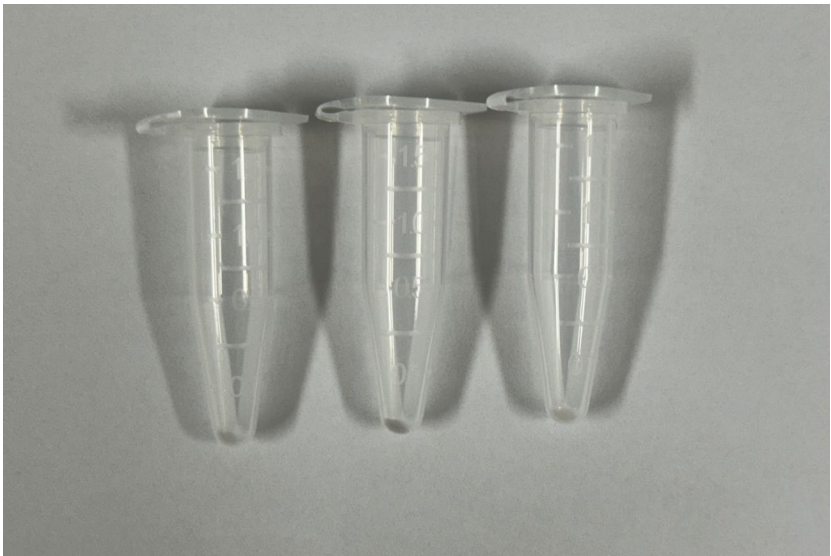

Supplement: Supplementary file 1 — Supplementary Material 1 [file 10103_2026_4908_MOESM1_ESM.pdf]
